# Effects of Varicella-Zoster Virus Glycoprotein E Carboxyl-Terminal Mutation on mRNA Vaccine Efficacy

**DOI:** 10.3390/vaccines9121440

**Published:** 2021-12-07

**Authors:** Han Cao, Yunfei Wang, Ning Luan, Kangyang Lin, Cunbao Liu

**Affiliations:** Institute of Medical Biology, Chinese Academy of Medical Sciences and Peking Union Medical College, Kunming 650118, China; caohan@imbcams.com.cn (H.C.); wangyf@imbcams.com.cn (Y.W.); luanning@imbcams.com.cn (N.L.); linky6679@163.com (K.L.)

**Keywords:** varicella zoster virus, glycoprotein E, carboxyl-terminal mutation, mRNA vaccine, cell-mediated immunity

## Abstract

Glycoprotein E (gE) is one of the most abundant glycoproteins in varicella-zoster virus and plays pivotal roles in virus replication and transmission between ganglia cells. Its extracellular domain has been successfully used as an antigen in subunit zoster vaccines. The intracellular C-terminal domain was reported to be decisive for gE trafficking between the endoplasmic reticulum, trans-Golgi network and endosomes and could influence virus spread and virus titers. Considering that the trafficking and distribution of mRNA vaccine-translated gE may be different from those of gE translated against the background of the viral genome (e.g., most gE in virus-infected cells exists as heterodimers with another glycoprotein, gI,), which may influence the immunogenicity of gE-based mRNA vaccines, we compared the humoral and cellular immunity induced by LNP-encapsulated mRNA sequences encoding the whole length of gE, the extracellular domain of gE and a C-terminal double mutant of gE (mutant Y569A with original motif AYRV, which targets gE to TGN, and mutants S593A, S595A, T596A and T598A with the original motif SSTT) that were reported to enhance virus spread and elevate virus titers. The results showed that while the humoral and cellular immunity induced by all of the mRNA vaccines was comparable to or better than that induced by the AS01B-adjuvanted subunit vaccines, the C-terminal double mutant of gE showed stable advantages in all of the indicators tested, including gE-specific IgG titers and T cell responses, and could be adopted as a candidate for both safer varicella vaccines and effective zoster vaccines.

## 1. Introduction

Ionizable lipid nanoparticle (LNP)-encapsulated mRNA vaccines have been approved by the FDA within one year of their development to prevent coronavirus disease 2019 (COVID-19), which is caused by severe acute respiratory syndrome coronavirus 2 (SARS-CoV-2) [1,2]. In addition to many other advantages, such as rapid development due to streamlined processes, low cost due to in vitro transcription and absence of antigen purification, SARS-CoV-2 mRNA vaccines have also shown a much better protection rate than other existing vaccine forms. In addition to the innate immune mobilization ability of mRNA itself for mRNA vaccines, its outstanding performance is also attributed substantially to the unique mechanism of intracellular translation of antigens: the protein translated from the mRNA that enters the cell of the vaccinated person has a high fidelity of posttranslational modifications, such as glycosylation, which is vital for the correct spatial structure of protein antigens; encoded and secreted protein antigens can be processed and presented by major histocompatibility complex (MHC) class II to stimulate CD4+ T helper cells to induce humoral immunity. Most importantly, cell-mediated immunity (CMI), which is less efficiently induced by subunit or inactivated forms of antigens and is highly dependent on the limited available adjuvants, could also be achieved incidentally by the self-adjuvant character of mRNA itself and the mechanism of mRNA vaccine antigen production: the protein antigens translated by mRNA in the cytoplasm could be fully processed into polypeptides and presented to MHC I as heterologous antigens produced by viral infection, which will activate CD8+ cytotoxic T lymphocytes that execute cellular immunity to selectively eliminate cells that express foreign antigens, such as virus-infected host cells [3].

CMI plays decisive roles in the efficacy of zoster vaccines [4,5,6,7,8,9]. As its name indicates, varicella-zoster virus (VZV) causes two different diseases: varicella/chicken pox upon primary infection and zoster/shingles when latent viruses in the sensory ganglia reactivate [10]. In fact, nearly every individual comes into contact with VZV before adulthood because of its high infectivity. While attenuated vaccines based on the Oka strain could effectively prevent infection with wild strains that cause varicella through humoral immunity, these vaccine strains could also lurk in the sensory ganglia and reactivate as wild strains do to cause zoster when the immune system is senescent (e.g., aging) or compromised (e.g., during infection by HIV or clinical immunosuppressive treatment) [11,12,13,14]. While pre-existing cellular immune responses upon primary VZV exposure or varicella vaccination could be boosted by the Oka strain at a dose as high as 19,400 plaque-forming units (PFU, compared with 1000–10,000 PFU in varicella vaccines), the attenuated zoster vaccine ZOSTVAX^R^ (developed by Merk in 2005) showed a much lower protection rate than the VZV glycoprotein E (gE) and AS01B adjuvanted subunit vaccine Shingrix^TM^ (developed by GSK in 2017) [15,16,17,18,19,20]. Considering the high price (approximately USD 150–200 per dose and two doses needed) of Shingrix^TM^, which is attributed mainly to the limited supply of its adjuvant component QS21, more economical zoster vaccines without a production limit are still needed, and mRNA vaccines with the advantages mentioned above are potential candidates [21,22].

In fact, a mRNA form of the zoster vaccine has been tested in nonhuman primates and showed comparable efficacy to that induced by VZV gE and AS01B adjuvanted subunit vaccines [23]. gE is one of the most abundant glycoproteins in VZV and plays pivotal roles in virus replication and transmission between ganglia cells [24,25,26,27]. As a highly glycosylated type I membrane protein, it frequently travels between the endoplasmic reticulum (ER), trans-Golgi network (TGN) and endosomes [28,29,30]. Although the extracellular domain was used as an antigen in Shingrix^TM^ subunit vaccines, mainly to lower the difficulties of protein purification, the intracellular carboxyl terminus of gE was decisive for gE trafficking and should be evaluated in mRNA vaccines, which depend on antigen production in vivo [31,32,33]. Considering that the trafficking and distribution of mRNA vaccine-translated gE may be different from those translated against the background of the viral genome (e.g., most gE in virus-infected cells exists as heterodimers with another glycoprotein, gI), we compared the immunity induced by LNP-encapsulated mRNA vaccines encoding gE with several C-terminal domain mutations that were reported to affect the translocation of gE in different parts of cells, hoping to find a more suitable gE sequence that induces better CMI for zoster vaccines.

## 2. Materials and Methods

### 2.1. Preparation of Vaccines

DNA sequences encoding the whole length of gE (gE, 623 aa), the extracellular domain (1–538 aa) of gE (gE-E) and the C-terminal double mutant (mutant Y569A with original motif AYRV, which targets gE to TGN, and mutants S593A, S595A, T596A and T598A with original motif SSTT, which targets gE to the TGN or plasma membrane) of gE (gE-M) were codon-optimized and synthesized with 5′ and 3′ untranslated regions (Sangon Biotech Co., Ltd., Shanghai, China) [23,31,34,35]. After linearization, T7 polymerase-mediated transcription was performed using an mRNA synthesis kit (APEXBIO Technology, Houston, TX, USA), and the product was purified with Monarch^R^ RNA purification columns (NEW ENGLAND BioLabs Inc., Ipswich, MA, USA).

LNP vaccines were prepared using a modified procedure previously described for mRNA vaccines [36,37,38]. Briefly, lipids (from AVT Pharmaceutical Technology Co., Ltd., Shanghai, China) were dissolved in ethanol at molar ratios of 50:10:37.5:2.5 (MC3: DSPC: cholesterol: DMG-PEG2000). The lipid mixtures were combined with 100 mM citrate buffer (pH 4.0) containing the above mRNA at a ratio of 3:1 with a microfluidic mixer (Precision Nanosystems, Inc., Vancouver, BC, Canada). Formulations were dialyzed against PBS, concentrated with a centrifugal filtration tube (Millipore, Tullagreen, Carrigtwohill, Co. Cork, Ireland), passed through a 0.22 μm syringe filter (PALL) and stored at 4 °C until use. Particle sizes were tested with a Zetasizer Nano ZS particle size analyzer (Malvern Panalytical, Malvern, UK). Loaded mRNA was detected with both a 1% denatured agarose gel and a Quant-iT^TM^ RiboGreen^R^ RNA Reagent Kit (Thermo Fisher, Eugene, OR, USA). The encapsulation efficiency was calculated as the amount of loaded nucleic acids detected compared with the amount of initial nucleic acids input in citrate buffer.

### 2.2. Animal Studies

Six-week-old female specific pathogen-free (SPF) C57BL/6N mice (15–18 g) were purchased from Vital River Laboratory Animal Technology Ltd. (Beijing, China), randomly divided into groups of 6 mice each (*n* = 6), maintained under SPF conditions and housed with free access to food and water at the Central Animal Services of the Institute of Medical Biology, Chinese Academy of Medical Sciences (IMB, CAMS). Polyinosinic–polycytidylic acid (Poly I:C, from InvivoGen, Inc., San Diego, CA, USA) and alum (Thermo Fisher, Eugene, OR, USA) mixtures or AS01B (from GSK, MD, USA)-adjuvanted extracellular domain of gE were used as controls, and PBS was used as a blank control. The mice were immunized intramuscularly in the thigh muscle twice with 50 μL of immunogen at 3-week intervals. After anesthetization by intraperitoneal injection of tribromoethanol, blood samples (via cardiac puncture) and spleens were collected 2 weeks after the final immunization for further analysis.

### 2.3. Enzyme-Linked Immunosorbent Assay (ELISA) of Antibody Titers

After clotting at 4 °C overnight, serum was collected after centrifugation at 3000 rpm for 10 min. gE-specific IgG titers were detected by ELISA, as previously described [39].

### 2.4. Enzyme-Linked Immunospot Assay (ELISPOT) of Splenocytes

Female spleens were dispersed with a 70 μm cell strainer (BD, USA). After red cell lysis by ammonium–chloride–potassium (ACK) lysis buffer at room temperature for 5 min, splenocytes were resuspended in Roswell Park Memorial Institute (RPMI) 1640 medium with 10% *v/v* fetal bovine serum (both from Biological Industries, Israel) and penicillin–streptomycin (Thermo Fisher) at a final concentration of 3 × 10^6^ cells/mL. Then, 100 μL of cells was added to each well of a 96-well plate (Corning, USA) for further analysis with an ELISPOT assay kit (BD) according to the manufacturer’s protocol. The extracellular domain of gE expressed in Chinese hamster ovary cells (supplied by AtaGenix Laboratory Co., Ltd., Wuhan, China) at 20 μg/mL was added to stimulate gE-specific T cell responses by incubation overnight. Spots were counted with an ELISPOT reader system (Autoimmun Diagnostika GmbH, Germany) after immunoimaging.

### 2.5. Flow Cytometry

The splenocytes prepared as described above were also analyzed by flow cytometry to determine the proportion of activated or memory T cells [40]. Briefly, 10 μg/mL extracellular domain of gE was incubated for 2 h, and brefeldin A was added and incubated overnight to block cytokine release. After the addition of 5 μg/mL CD16/CD32 antibodies to block the nonspecific binding of Fc receptors by incubation at 4 °C for 10 min, PerCP-tagged anti-mouse CD4, FITC-tagged anti-mouse CD8a, Brilliant Violet 421-tagged anti-mouse CD62L and Brilliant Violet 510-tagged anti-mouse CD44 antibodies were added and incubated at 4 °C for another 30 min. After washing with permeabilization wash buffer, PE-tagged anti-mouse IFN-γ and APC-tagged anti-mouse IL-2 antibodies were added and incubated in the dark at room temperature for 30 min. Cells were gated (FSC/SSC), and samples with more than 20,000 events of CD4+ or CD8+ T cells were analyzed with a CytoFLEX flow cytometer (Beckman, Indianapolis, IN, USA) and FlowJo software (BD, USA).

### 2.6. Statistical Analysis

Data were analyzed with one-way analysis of variance (ANOVA) followed by Dunnett’s multiple comparisons test, with the gE-M mRNA vaccine group as the control. GraphPad Prism 8.0 (GraphPad Software Inc., La Jolla, CA. USA) was used for statistical analyses.

## 3. Results

### 3.1. LNPs Efficiently Encapsulated mRNA Antigens with Uniform Particle Sizes

**Figure 1:**
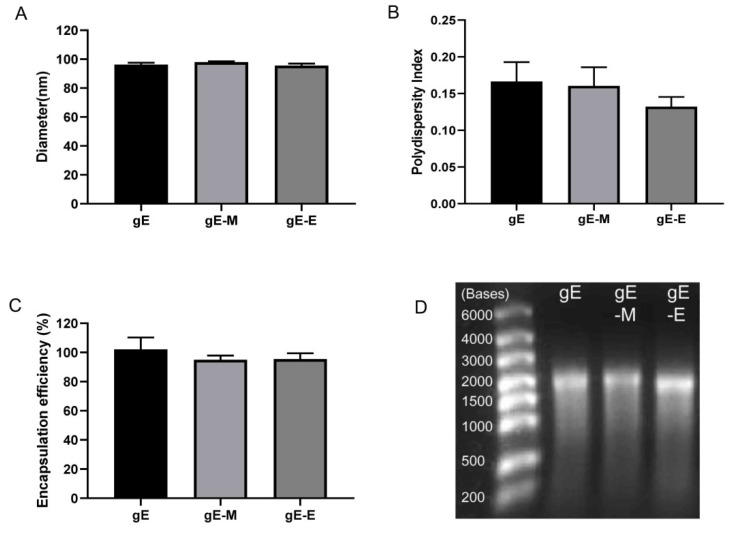
Characterization of LNP mRNA vaccines. (**A**) Diameters tested by size analyzer; (**B**) polydispersity index of LNPs; (**C**) mRNA encapsulation efficiency; (**D**) loaded mRNA detected with a 1% denatured agarose gel.

The diameters of LNPs encapsulating gE, gE-M and gE-E mRNA were 96.30 nm, 97.92 nm and 95.52 nm, respectively (Figure 1A). All of the polydispersity indices (PDIs), which are measures of the heterogeneity of a sample based on size, were lower than 0.2 (0.166 for gE, 0.160 for gE-M and 0.132 for gE-E, Figure 1B), which indicates good uniformity of the particle sizes. When 100 μg of mRNA was added to 1.5 mL of citrate buffer as the raw material for 12 doses of LNP vaccine, the encapsulation efficiencies were all above 94% (102.05% for gE, 94.91% for gE-M and 95.45% for gE-E, Figure 1C). All of these encapsulated mRNAs showed good integrity on denatured agarose gel as bands at approximately 2000 bases, with gE-E being slightly lower (Figure 1D).

### 3.2. mRNA Vaccines with C-terminal Mutations (gE-M) Induced the Most Potent Humoral Immune Responses

**Figure 2:**
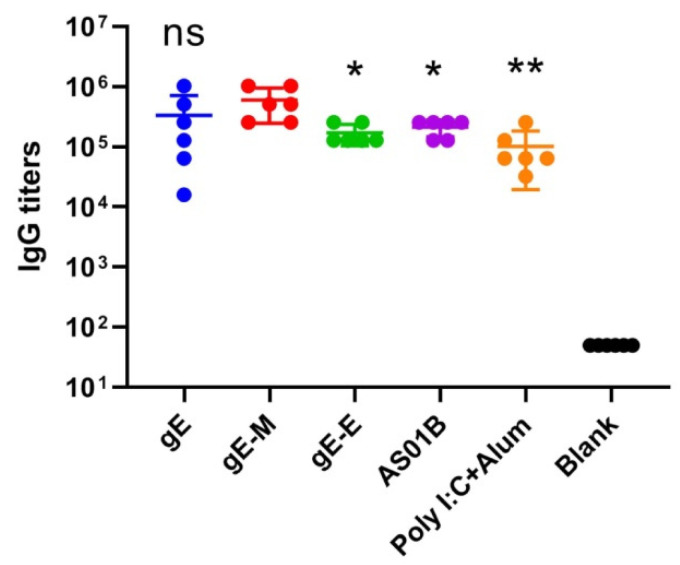
gE-specific IgG titers detected by enzyme-linked immunosorbent assay (ELISA). IgG titers were compared using one-way analysis of variance (ANOVA) followed by Dunnett’s multiple comparisons test, with the gE-M group as a control. * *p* < 0.05, ** *p* < 0.01. ns, no significant difference.

For the two control subunit vaccines, each dose of the AS01B group contained 5 μg of gE and 50 μL of AS01B adjuvants, which equals 1/10 dose of Singrix^TM^, and each dose of the Poly I:C+Alum group contained 10 μg of gE and 15 μg of Poly I:C. The gE-specific IgG titers of the AS01B group (213,333) were approximately two times those of the Poly I:C+Alum group (101,333) and higher than those of the gE-E group (170,667), but the mRNA vaccine with C-terminal mutations (gE-M group) showed the highest gE-specific IgG titers (597,333), which were approximately two times those of the whole gE group (gE, 333,333) and three times those of the AS01B group (Figure 2, *p* < 0.05).

### 3.3. C-Terminal Mutations (gE-M) Were Slightly Helpful in Inducing CMI in mRNA Vaccines

**Figure 3:**
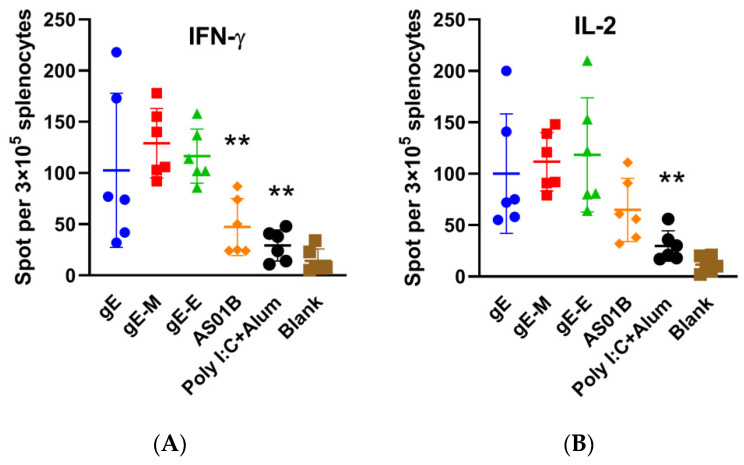
Enzyme-linked immunospot assay (ELISPOT) of splenocytes. (**A**) IFN-γ-producing splenocytes after gE stimulation; (**B**) IL-2-producing splenocytes after gE stimulation. ELISPOT numbers were compared using one-way ANOVA followed by Dunnett’s multiple comparisons test, with the gE-M group as a control. ** *p* < 0.01.

ELISPOT analysis showed that the mRNA vaccine with C-terminal mutations (gE-M group) showed the most IFN-γ-producing splenocytes (129 spots per 3 × 10^5^ splenocytes in Figure 3A) among all three mRNA vaccines (116.5 for gE-E and 102.7 for gE) after gE stimulation, and the value was significantly higher than that of the AS01B group (47.3, *p* < 0.01). For gE-specific IL-2-producing splenocytes (Figure 3B), the gE-M group (spots of 111.7) had a slightly higher value than the gE group (spots of 100.2) but a slightly lower value than the gE-E group (spots of 118.3). Considering the higher outlier data in the gE-E group, we conclude that C-terminal mutations may still be the most potent in inducing CMI in mRNA vaccines.

**Figure 4:**
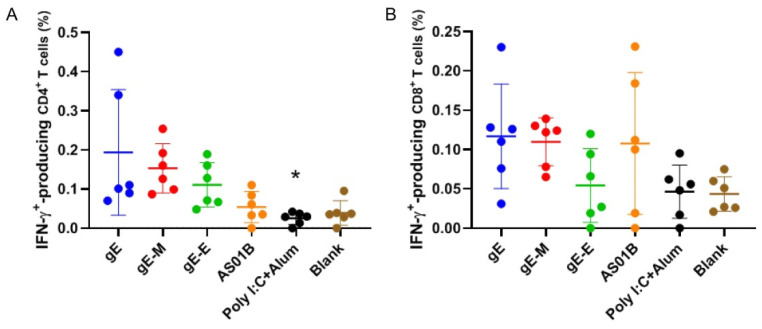
Flow cytometry assay for gE-specific IFN-γ-producing T cells. (**A**) Proportion of IFN-γ-producing CD4+ T cells among splenocytes after stimulation with gE; (**B**) proportion of IFN-γ-producing CD8+ T cells among splenocytes after stimulation with gE. Data were analyzed using one-way ANOVA followed by Dunnett’s multiple comparisons test, with the gE-M group as a control. * *p* < 0.05.

Similar conclusions could be drawn from the results of the flow cytometry analysis (Figure 4). While the gE-M group showed higher proportions of IFN-γ-producing CD4+ (Figure 4A) and IFN-γ-producing CD8+ T cells (Figure 4B) compared with the gE-E group, these values were slightly lower than those of the gE group concerning IFN-γ-producing CD4+ (0.153% versus 0.194%) and IFN-γ-producing CD8+ (0.110% versus 0.117%) T cells when the higher outlier data in the gE group were included.

### 3.4. C-Terminal Mutations (gE-M) Were Slightly Helpful to Elevate T Cell Memory in mRNA Vaccines

**Figure 5:**
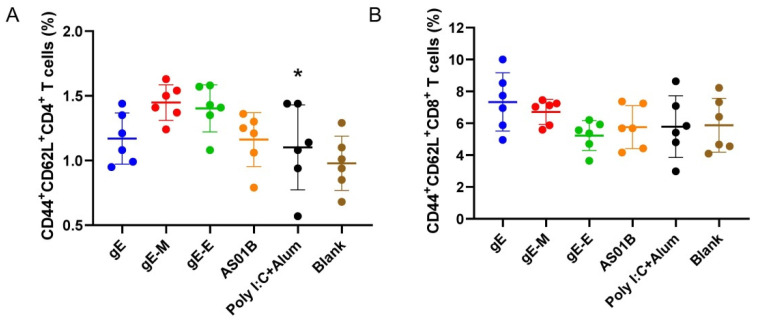
Flow cytometry assay for memory T cells. (**A**) Proportion of memory CD4+ T cells among splenocytes after stimulation with gE; (**B**) proportion of CD8+ memory T cells among splenocytes after stimulation with gE. Data were analyzed using one-way ANOVA followed by Dunnett’s multiple comparisons test, with the gE-M group as a control. * *p* < 0.05.

Antigen-experienced (CD44+) T cells responsible for central memory (CD62L+) were analyzed by flow cytometry [41,42]. Although the gE-M group showed the highest proportion of CD4+ central memory cells (1.45%) among all the vaccine groups (Figure 5A), a significant difference was detected only compared with the Poly I:C+Alum group (1.10%, *p* < 0.05). For CD8+ central memory cells, the gE group was the highest (7.34%), followed by the gE-M group (6.72%), but no significant difference existed between gE-M and any other group (Figure 5B).

## 4. Discussion

As one of the most abundant glycoproteins with conserved neutralization epitopes and T cell epitopes, gE is essential for VZV replication and transmission between ganglia cells [24,25,26,27,43]. As a subunit antigen, the extracellular domain has shown potential as a safe varicella vaccine that could avoid the latency of viruses from live-attenuated vaccines, which may reactivate as herpes zoster, and has potential as a zoster vaccine with suitable adjuvants that induce powerful CMI [9,36,44,45]. Although the subunit vaccine Shingrix^TM^ with the extracellular domain of gE adjuvanted with the AS01B system showed an excellent protection rate against herpes zoster, it was not reported for use as a safer varicella vaccine, mainly because of the limited supply and infeasible synthesis of the key components of AS01B, i.e., the polysaccharide mixture QS21, which is extracted from the bark of *Quillaja saponaria*, which is distributed in the temperate regions of South America [21,22].

LNP-encapsulated mRNA vaccines could induce both humoral immunity and CMI by the in vivo translation of protein antigens and their self-adjuvant character [3]. Theoretically, the extracellular domain of VZV gE could be used as a corresponding mRNA vaccine. Notably, the intracellular carboxyl terminus of gE was found to be decisive for gE trafficking between the ER, TGN and endosomes [28,29,30]. Considering that the distribution of gE after translation may influence the final presentation pathway of gE as antigens, whether carboxyl-terminal mutations may influence the final immunity of mRNA vaccines should be evaluated in pursuit of better immunity [23].

When encapsulated in LNPs, all mRNA sequences, including the extracellular domain (1–538 aa) of gE, that were used in subunit vaccines to lower the difficulties of protein purification (gE-E), the whole sequence of gE (gE, 623 aa) and C-terminal double mutants (mutant Y569A and mutants S593A, S595A, T596A and T598A) of gE (gE-M), which were reported to enhance virus spread and increase virus titers against the background of the viral genome, showed uniform characteristics, including diameter, PDI and encapsulation efficiency (Figure 1). Two weeks after two intramuscular immunizations at 3-week intervals at a dose of approximately 7.5 μ/dose, gE-M induced the most potent humoral immune responses, showing the highest gE-specific IgG titers (Figure 2), which indicated that gE-M is usable as a varicella vaccine. gE-M also showed stable advantages in all of the indicators tested for CMI that play decisive roles in the efficacy of zoster vaccines, including gE-specific IFN-γ/IL-2-producing splenocyte numbers analyzed by ELISPOT (Figure 3) and the proportion of gE-specific IFN-γ-producing CD4+/CD8+ T cells among splenocytes analyzed by flow cytometry analysis (Figure 4) [4,5,6,7,8,9].

Although not statistically significant, the gE-specific IFN-γ produced CD4+ T cells, which are more frequently adopted than CD8+ T cells as good indicators for the potential of zoster vaccines in animal experiments and clinical trials, at approximately 2.84 times the quantity of the AS01B adjuvanted subunit vaccines, which was consistent with the result of a 573 aa carboxyl-terminal truncated and Y569A-mutated gE mRNA vaccine tested in nonhuman primates [17,23,44]. Because we adopted nearly identical nucleic acid sequences derived from the whole length of gE for each mRNA vaccine, the advantages of gE-M-induced immunity may be attributed mainly to the influence of carboxyl-terminal mutations and the distribution of translated protein antigens, as observed against the background of the viral genome, which ultimately manifested as rapid virus spread and higher virus titers [31]. Interestingly, gE-M also showed stable advantages in both CD4+ and CD8+ T cell memory indicators, as shown by a higher proportion of antigen-experienced (CD44+) central memory (CD62L+) T cells among splenocytes observed by flow cytometry analysis (Figure 5).

## 5. Conclusions

In conclusion, while mRNA vaccines encoding the whole length of gE, the extracellular domain of gE and a C-terminal double mutant sequence of gE all showed comparable or better humoral and cellular immunity to the AS01B adjuvanted subunit vaccines, the vaccines encoding double mutants at the C-terminus, which were reported to enhance virus spread and higher virus titers against the background of the viral genome, showed stable advantages in all of the indicators tested for humoral immunity and CMI and could be adopted as both safe varicella and effective zoster mRNA vaccine candidates.

## Data Availability

All data used during the study are available from the corresponding author by request.
